# Perception of e-professionalism of doctors of medicine and doctors of dental medicine among the general population in Croatia

**DOI:** 10.1186/s12910-026-01427-1

**Published:** 2026-02-28

**Authors:** Marko Marelić, Joško Viskić, Kristijan Sedak, Marjeta Majer, Tea Vukušić Rukavina

**Affiliations:** 1 Andrija Štampar School of Public Health, School of Medicine, University of Zagreb, Rockefeller Str. 4, Zagreb, 10 000 Croatia; 2 Department of Fixed Prosthodontics, School of Dental Medicine, University of Zagreb, Zagreb, Croatia; 3 Department of Communication Sciences, Catholic University of Croatia, Zagreb, Croatia

**Keywords:** Social media, E-professionalism, Medicine, Dental medicine, Medical doctors, Dental medicine doctors, General population

## Abstract

**Background:**

Health care professionals’ (HCP) behaviour on social media (SM) can affect the perception that their colleagues, patients, and the general population have about them and their professionalism. There has been extensive research on the perception of HCPs’ e-professionalism within the student HCPs population and the practising HCPs, however, the research on perceptions of the general population has been missing so far. This study had three distinct aims: (1) to examine the perception of medical doctors (MDs) and doctors of dental medicine (DMDs) e-professionalism among the general population in Croatia using the e-Professionalism Assessment Compatibility Index (ePACI) index, (2) to investigate tendencies and differences regarding values of ePACI index among the general population in Croatia, (3) to determine which sociodemographic characteristics are predictors of the ePACI index results among the general population.

**Methods:**

The research is conducted on a probabilistic representative sample of the Croatian general population. To evaluate the perception of MDs’ and DMDs’ e-professionalism among the general population in Croatia, the ePACI index was used. Principal component analysis was used to check the dimensionality of the items in the ePACI instrument. To test multivariate relationships, a hierarchical regression analysis was used.

**Results:**

A total of 1,000 responses were collected, of which 788 participants entered the analysis. Results showed general population in Croatia holds a “conservative” perception of the professionalism of doctors’ posts on SM (t_787_=28.022, *p* < 0.001). Hierarchical regression analysis revealed that age is the only significant predictor of the ePACI index for the general population (β = 0.131, t = 2.755, *p* = 0.006), with older individuals demonstrating a more “conservative” perception of the doctors’ posts on SM.

**Conclusions:**

The general population in Croatia holds a strict or “conservative” perception of doctors’ e-professionalism. This suggests that a significant number of posts on the MDs’ and DMDs’ profiles might be perceived as unprofessional. Given that the general population is more inclined to perceive posts as unprofessional compared to MDs and DMDs themselves, there is a need for continuous and focused education for doctors to maintain their e-professionalism that also includes the perception of the general population.

**Supplementary Information:**

The online version contains supplementary material available at 10.1186/s12910-026-01427-1.

## Background

Professional behaviour on social media (SM) is a towering concern for modern society. As more and more daily interactions and communications have transferred to SM, professionalism has been tested and compromised through all primary professions in contemporary society [1]. In particular, healthcare professionals (HCPs) have encountered several barriers that lead to moral, ethical and legal reprimand and resulted in public outrage and legal consequences [2, 3].

As traditional concepts of professionalism still apply at the individual, interpersonal and societal levels, *e-professionalism* has emerged as a new term, attempting to encompass the nuances of online professional behaviour. E-professionalism was defined by Cain et al. as “attitudes and behaviours (some of which may occur in private settings) reflecting traditional professionalism paradigms manifested through digital media” [4].

The scientific community has also encountered problems in navigating the changing social landscape. As society has been evolving rapidly in understanding moral and professional boundaries, outdated methods and instruments have been forced to change and upgrade better to include the diverse world, especially the digital world, we live in today. The best example of such changes was the #medbikini movement in 2020 [5–7] and the subsequent evolution and development of the contemporary coding scheme (SMePROF coding scheme) for the assessment of unprofessional behaviour on SM published by Vukušić Rukavina et al. in 2022 [8].

There has been extensive research on the perception of professional and unprofessional behaviour on SM within the student HCPs population and the practising HCPs [9–15]. As student-patient interactions increase, the opportunity for unprofessional behaviour to be posted on SM and seen by the general public increases [10, 16, 17]. In their study of a variety of HCP students, White et al. were among the first to present a precise and straightforward questionnaire with examples of SM posts that can be evaluated for professionalism [11]. Viskić et al. and Relić et al. more recently investigated a similar population (medical and dental students in Croatia) using an adapted and validated version of White’s questionnaire and found differences in professionalism perception between the investigated groups [13, 18]. In addition to these differences in perception, other research suggests that attitudes toward e-professionalism also vary between medical and dental students [19].

Within the population of practising HCPs, results are similar to those in the student body [8, 20–22]. Practising HCPs perceive transgressions in professional behaviour very clearly, with posts containing illegal activities, clear Health Insurance Portability and Accountability Act (HIPAA) violations and elicit sexual behaviour as the ones with the most unprofessional perception [21]. A novel way of evaluating e-professionalism developed based on findings from White et al. [11] and Vukušić Rukavina et al. [8], was the E‑professionalism assessment compatibility index (ePACI). It was devised by Viskić et al. as a tool for assessing the deviations of the e-professionalism perceptions from the norm [21]. This research devised a scientifically and theoretically justified way of defining “the correct answer”, or “the norm” [21], using the SMePROF rubric [8] to assess unprofessional content for each item. The results of this research present that both medical doctors (MDs) and dental doctors (DMDs) have a “conservative” or more cautious perception post on SM, compared to “the norm”, which can lead to apprehension in usage and potentially lead to these groups not utilising the full potential of SM in all the positive ways that have been identified [23]. The SM usage can improve patients’ support [20, 24] and knowledge [25] for certain health-related aspects. However, interactions with doctors on the SM can also affect their perceptions of doctors’ professionalism and credibility [26]. So far, most of the scientific literature has been investigating the perception of e-professionalism among HCPs themselves [9–14, 21–23, 27, 28]. However, an extensive knowledge gap exists in the general population’s perception of HCPs’ professionalism on SM [20, 21, 26]. Recently, Freire et al. investigated the influence of the use of dental practice SM on patients when they last changed their provider [29]. The public’s perception of e-professionalism and credibility is essential for the comprehensive perspective needed for the further development of guidelines regarding the e-professionalism of HCPs [30]. Therefore, we have designed a study with the study objects, the general population of Croatia, that represent potential or current patients, to gain more insight into the perception of MDs’ and DMDs’ e-professionalism among the general population in Croatia.

### Aim

This study had three distinct aims:

1. to examine the perception of MDs’ and DMDs’ e-professionalism among the general population in Croatia using the ePACI index,
2. to investigate tendencies and differences regarding values of ePACI index among the general population in Croatia,
3. to determine which sociodemographic characteristics are predictors of the ePACI index results among the general population.

## Methods

### Design

The collection of data was carried out by the Ipsos Agency as part of the research called “Omnibus” [31]. An omnibus survey is a quantitative research method where data on various subjects is collected during the same interview [32]. Omnibus is a field survey that is conducted using the face-to-face method, and which the Ipsos Agency conducts every month. Omnibus questions related to the demographic characteristics of respondents, and specific instruments, including the instrument for measuring e-professionalism perception that allows the creation of the ePACI index.

The instrument for measuring perception of e-professionalism was approved by the ethical boards of the University of Zagreb School of Medicine (641–01/18–02/01) and the University of Zagreb School of Dental Medicine (05-PA-24-2/2018). Since this research was conducted as a part of the omnibus research conducted by the Ipsos Agency, each participant was provided with informed consent and signed a consent form before completing the survey. Participants could withdraw from the study at any point and had the right not to answer any questions in the survey. IPSOS Agency is certified with ISO 20252:2012 for market research, media, and public opinion research and must comply with Regulation (EU) 2016/679 of the European Parliament and Council when conducting the research.

### Study instrument

The instrument used to measure the perception of e-professionalism was originally created by White et al. and contained 19 items [11]. Viskić et al. [21] translated this instrument into Croatian and modified it by removing two items that were only relevant to students. Viskić et al. [21] also developed the ePACI index based on responses from the adapted White et al. instrument [11].

The ePACI index works as an additive measure with a specific scoring system: correct answers receive 0 points, considering acceptable behaviour as unprofessional earns + 2 points (leaning toward professional content), and viewing unprofessional behaviour as acceptable receives − 1 point (leaning toward unprofessional content). Professional items are weighted more heavily to balance the uneven distribution of items (10 unprofessional versus 5 professional).

The final index is standardized to range from − 1 to + 1. Negative values indicate a “liberal” tendency, positive values show a “conservative” tendency, and values near zero reflect perceptions that align with established norms [21]. It’s worth noting that the terms “liberal” and “conservative” are used descriptively without any positive or negative judgment [21]. For a complete explanation of the mathematical and statistical methodology, readers can refer to Viskić et al. [21].

### Sampling process of the respondents

A probabilistic representative sample of Croatian general population was obtained using the multi-stage sampling. A two-way stratification was performed according to the two characteristics: six traditional regions that are defined as a set of existing counties and the size of the settlement (grouped in four sizes: up to 2,000 inhabitants, 2,001–10,000 inhabitants, 10,001–100,000 inhabitants and more than 100,000 inhabitants).

The multi-stage procedure for selecting respondents in the sample was as follows. In the first stage, the probability proportional to size (PPS) method was used to choose settlements as primary selection units and the probability of selecting a unit was proportional to the size of the settlement (size is defined according to the number of inhabitants aged 15 and over). The largest cities in all regions were necessarily included in the sample. In the second stage, a random selection of households was conducted as secondary selection units based on the random selection of addresses for a given number of starting points (random starting points method) and households were selected using the random walk method. In the third stage, a person was selected randomly as the final selection unit, within the household, according to a predetermined quota by sex and age.

The planned sample size was 1000 respondents aged 16 years or older, which is more than sufficient based on standard sample size calculations for the adult population of Croatia, which require significantly fewer cases to achieve a 95% confidence level with a 5% margin of error (approximately 400 respondents). The sample was intentionally set at 1000 not only to ensure robust overall estimates but also to allow for meaningful subgroup analyses.

### Data collection and analysis

Exclusively, interviewers who were instructed for the needs of the project carried out data collection. The regional coordinators instructed the interviewers in writing and, if necessary, orally. All interviewers underwent detailed training on the basic rules and principles of conducting field research using the personal interview method before participating in this project.

An integral part of data collection is the control of the interviewer’s work. The control covered a minimum of 15% of the conducted interviews with each interviewer.

Possible deviations of the sample structure from the population structure are eliminated by the post-stratification process (weighting) according to sex, age, and education, with the following data from the 2011 Census and available data from the 2021 Census [33, 34]. The survey was conducted between July 1 and July 19, 2022.

Demographic data were analysed using descriptive statistics. Comparisons of the groups were calculated using the Kruskal–Wallis test (with the Tamhane post-hoc test) and the Mann–Whitney U test. Correlations were explored using Pearson or Spearman correlation coefficients. Inter-item correlations were explored using Pearson correlation coefficient, while for dichotomous variables, point-biserial correlation coefficients with quantitative variables and phi coefficients of association between two dichotomous variables were calculated. The normality of the distribution for continuous variables was assessed using the Kolmogorov–Smirnov test. Principal component analysis was used to check the dimensionality of the items in the ePACI instrument. To test multivariate relationships, a hierarchical regression analysis was used, in which the dependent variable was the ePACI index and the predictors were the sociodemographic characteristics of the respondents. Data processing and analysis were performed using the IBM SPSS Statistics 26 statistical package.

## Results

A total of 1,000 responses were collected, of which 788 participants responded to all 17 items on the perception of unprofessional content instrument, which is of central importance to this study. Therefore, the results of the perception of unprofessional content and sociodemographics are investigated on a sample of 788 responses. For the purposes of hierarchical regression analysis, the sample consists of 653 participants because participants who did not respond to the question about personal income were excluded.

The sociodemographic characteristics of the sample and differences between the groups are shown in Table 1. The sex distribution in the sample is mostly equal, with a female proportion of 52.1%. The mean age of the sample was 48 years, with a minimum of 16 and a maximum of 89 years. About two-thirds of the respondents live in cities and one-third in villages. About a third of all respondents live in cities larger than 75,000 inhabitants. It’s important to note that 75% of the respondents use the internet daily and about 18% of respondents do not use the internet at all.

**Table 1:** The sociodemographic characteristics of the sample and differences between groups on the ePACI index (*N* = 788)

Variables	Descriptives	ePACI		Statistical test
	*N* (%)	Mean	SD	
Sex				
Male	377 (47.9)	0.58	0.608	U = 92,461, *p* = 0.776^a^
Female	410 (52.1)	0.61	0.580	
Age				
Mean	47.97	N/A	N/A	r_s_ =0.251, *p* < 0.001^b^**
Standard deviation	18.089	N/A	N/A	
Type of settlement				
Village	288 (36.6)	0.51	0.682	U = 81584.5, *p* = 0.015^a^*
City	500 (63.4)	0.64	0.531	
Size of the settlement				
Up to 2000 inhabitants^c^	288 (36.6)	0.51	0.682	χ2_3_=8.285, *p* = 0.040^d^*
2,001–10,000 inhabitants	127 (16.2)	0.63	0.598	
10, 001–75,000 inhabitants	158 (20.0)	0.61	0.534	
75,001 and more inhabitants^c^	214 (27.2)	0.68	0.483	
Education degree				
Elementary school or lower	184 (23.4)	0.58	0.635	χ2_2_=2.327, *p* = 0.802^d^
Vocational school	391 (49.6)	0.59	0.607	
Gymnasium	45 (5.8)	0.53	0.562	
Undergraduate study / First degree of the faculty (Bachelor)	67 (8.5)	0.57	0.603	
Graduate study / Faculty / Academy / College	86 (10.9)	0.66	0.453	
Postgraduate studies (specialist, master’s, PhD)	12 (1.5)	0.58	0.580	
Don’t know/Without answer	2 (0.3)	0.59	0.594	
Monthly income (*n* = 655)				
No personal income last month	71 (9.0)	0.49	0.644	r_s_=-0.011, *p* = 0.758^b^
Up to HRK 1,000 (132.72€)	16 (2.0)	0.56	0.553	
From HRK 1,001 (132.86€) to HRK 2,000 (265.45€)	47 (6.0)	0.48	0.736	
From HRK 2001 (265.58€) to HRK 3000 (398.17€)	76 (9.6)	0.74	0.462	
From HRK 3,001 (398.30€) to HRK 4,000 (530.89€)	96 (12.2)	0.60	0.641	
From HRK 4,001 (531.02€) to HRK 5,000 (663.61€)	99 (12.6)	0.49	0.694	
From HRK 5,001 (663.75€) to HRK 6,000 (796.34€)	71 (9.0)	0.59	0.478	
From HRK 6,001 (796.47€) to HRK 7,000 (929.06€)	49 (6.2)	0.72	0.481	
From HRK 7,001 (929.19€) to HRK 8,000 (1061.78€)	47 (6.0)	0.57	0.599	
From HRK 8,001 (1061.92€) to HRK 9,000 (1194.51€)	21 (2.6)	0.57	0.600	
9001 (1194.64€) to 10,000 (1327.23€) HRK	23 (2.9)	0.36	0.635	
10,001 (1327.36€) to 11,000 (1459.95€) HRK	10 (1.3)	0.45	0.553	
HRK 11,001 (1460.08€) to HRK 12,000 (1592.67€)	12 (1.6)	0.48	0.746	
12,001 (1592.81€) and more	17 (2.1)	0.56	0.679	
Don’t know/Refuse to answer	132 (16.8)	N/A	N/A	
Frequency of internet use				
Every day	595 (75.6)	0.55	0.609	r_s_ =-0.165, *p* < 0.001^b^**
Several times a week	33 (4.2)	0.73	0.488	
Once a week	10 (1.3)	0.88	0.227	
Several times a month	1 (0.1)	1.00	N/A	
Once a month	4 (0.5)	0.59	0.883	
Rarely	6 (0.8)	0.88	0.250	
I do not use internet	138 (17.6)	0.73	0.543	

*p < 0.05

**p < 0.001

^a^Mann–Whitney U test

^b^Spearman correlation coefficient

^c^The Tamhane post hoc-test showed a statistically significant difference between settlements with up to 2,000 inhabitants and those with 75,001 or more inhabitants

^d^Kruskal–Wallis’s test

Differences in the ePACI index on sociodemographic variables are shown in Table 1. There is a statistically significant positive correlation between age and the ePACI index (r_s_=0.251, *p* < 0.001), tested using Spearman rank correlation coefficient due to the non-normal distribution of the age variable, as indicated by the Kolmogorov–Smirnov test (D₇₈₈=0.077, *p* < 0.001). This suggests that older respondents had a more conservative perception of MDs’ and DMDs’ e-professionalism. Respondents living in cities had statistically significant larger values of the ePACI index (more “conservative”) than respondents living in villages (U = 81584.5, *p* = 0.015). The Kruskal-Wallis test showed that there are statistically significant differences in the ePACI index regarding the size of the settlement (χ2_3_=8.285, *p* = 0.040). More specifically, the Tamhane post-hoc test showed that the only significant difference is between the settlements of up to 2,000 inhabitants and settlements of 75,001 and more inhabitants (*p* = 0.008), where those in the larger settlements had a more “conservative” ePACI index. The Spearman coefficient of correlation showed that there is a significant negative association between the frequency of internet use and the ePACI index, where those who use the internet more frequently had smaller ePACI index values (more “liberal”) (r_s_=-0.165, *p* < 0.001). There were no significant differences in the ePACI index based on sex, education degree, or monthly income.

Results of the perception of unprofessional content and principal component analysis are shown in Table 2. The posts are ordered according to component loadings.

**Table 2:** Perception of unprofessional content and principal component analysis (*N* = 788)

	Considered unprofessional	Component^a^	
	*N* (%)	1	2
Posts disclosing information about a patient/client	710 (90.1)	**0.840**	0.208
Posts involving overt sexual content	693 (88.0)	**0.831**	0.241
Pictures of an individual clearly behaving drunkenly	678 (86.1)	**0.813**	0.316
Petty criminal activity	684 (86.8)	**0.807**	0.311
Swearing or foul language	678 (86.1)	**0.802**	0.294
Posts depicting illicit drug consumption	691 (87.7)	**0.791**	0.228
Obscene gestures in photos (the middle finger, etc.)	679 (86.2)	**0.787**	0.336
Posts containing partial nudity	668 (84.8)	**0.741**	0.418
Photos of a patient/client	675 (85.7)	**0.715**	0.317
Status updates describing substantial alcohol consumption at a party	630 (80.0)	**0.646**	0.446
Attitudes of superiority or condescending behaviour (assumed because of professional status)	621 (78.9)	**0.506**	0.497
Displaying your current relationship status	493 (62.6)	0.173	**0.810**
Making opinionated comments about controversial issues	545 (69.2)	0.228	**0.753**
Posts describing an interaction with a patient/client, while not revealing any identifying information	519 (66.5)	0.174	**0.735**
Displaying membership in online groups dealing with controversial issues	595 (75.5)	0.424	**0.674**
A picture of an individual having one alcoholic beverage	602 (76.5)	0.476	**0.639**
Endorsements of a pharmaceutical or health product without a conflict-of-interest disclosure	548 (70.7)	0.357	**0.578**

Loadings in bold indicate the highest component loading for each item (these being the most salient loadings for the interpretation of each component)

^a^PCA. Extraction Method: Principal Component Analysis. Rotation Method: Varimax with Kaiser Normalization

Posts disclosing information about a patient/client are perceived as unprofessional by 90.1% of the respondents, making it the highest unprofessionally perceived content in our research. Posts involving overt sexual content (88%) and posts depicting illicit drug consumption followed closely behind (87.7%). The content that is least perceived as unprofessional was displaying current relationship status (62.6%), followed by posts that describe an interaction with a patient/client, while not revealing any identifying information (66.5%).

The value of Kaiser–Meyer–Olkin (KMO) (0.964) and Bartlett test (χ2_136_=9718.119, *p* < 0.001) show that it is meaningful to perform PCA. PCA with varimax rotation and Kaiser normalization was conducted. A total of two components were extracted with 65.92% of the variance explained. The first component can be called “Seriously unprofessional posts”, it is formed by 11 items and explains 57.12% of the variance. The second factor explains 8.8% of the variance, it is formed by the remaining six items and can be called “Professional posts”.

**Fig. 1:**
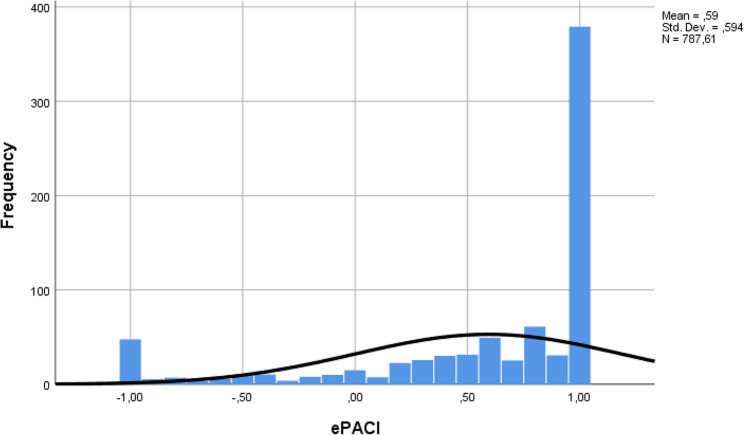
Histogram of distribution of ePACI values

The ePACI index was calculated as described by Viskić et al. [21] to investigate the perception of e-professional behaviour. The index ranged from − 1 to 1, with a mean of 0.59 (SD = 0.594). One sample t-test showed that responses in our sample statistically significantly deviated from the “correct” neutral answer, towards positive “conservative” values (t_787_=28.022, *p* < 0.001). Figure 1 shows a visual representation of the “conservative” deviation of ePACI values.

The Kolmogorov-Smirnov test showed that the ePACI index is not normally distributed (D_788_=0.246, *p* < 0.001), therefore, nonparametric measures are used to test the differences in the socioeconomic variables.

Inter-correlations between socioeconomic variables and direct correlations between the ePACI index and the predictors are shown in Table 3. The largest statistically significant correlation between predictors was that between the type of settlement and the size of the settlement (*r* = 0.854, *p* < 0.001). Other noteworthy significant positive correlations were between the level of education and the income level (*r* = 0.471, *p* < 0.01), and the use of the internet and the level of education (*r* = 0.341, *p* < 0.01). Statistically significant negative correlation is found between age and the use of the internet (*r*=-0.579, *p* < 0.001).

**Table 3:** Intercorrelations of variables in the regression model (r) (*N*=653^a^)

	1	2	3	4	5	6	7
1. ePACI	1						
2. Type of settlement^b^	0.107**	1					
3. Size of the settlement	0.111**	0.854**	1				
4. Sex^b^	0.043	0.047	0.057	1			
5. Age	0.176**	0.006	-0.040	0.085*	1		
6. Level of education	0.015	0.219**	0.256**	-0.019	-0.142**	1	
7. Income level	-0.007	0.167**	0.191**	-0.144**	-0.081*	0.471**	1
8. Use of the internet	-0.142**	0.090*	0.117**	-0.018	-0.579**	0.341**	0.255**

*p<0.05

**p<0.01

^a^ The *N* is 653 because only respondents who answered all items were included in the analysis

^b^ Dichotomous variable. Point-biserial correlation coefficients with quantitative or ordinal variables are presented, as well as phi coefficients of association between two dichotomous variables

There was a statistically significant positive correlation between the index and the type of settlement; size of settlement and age; and a statistically significant negative correlation between the use of the internet and the ePACI index. This means that those living in cities had more “conservative” scores on the ePACI index than those living in villages, as did the respondents living in larger settlements compared to the smaller ones. The older the respondents were, and the less they used the internet, the more conservative their ePACI index was. The correlation between the ePACI index and variables: sex, level of education and income, was not statistically significant.

The results of the hierarchical regression analysis are shown in Table 4. Prior to conducting hierarchical regression analyses, key assumptions were verified. Diagnostic checks confirmed satisfaction of key hierarchical regression assumptions: residual plots indicated linearity and homoscedasticity, and multicollinearity was low (max VIF = 3.80) and condition indices were acceptable (max condition index = 23.47). The predictors enter the regression analysis using the *enter* method and are separated into two steps. The logic of the order of inclusion of predictors in the model starts from what is “closer” to the respondents, i.e., what is given to them (socio-demographic variables) and goes towards what is “further” from them, i.e., learned or adopted.

**Table 4:** Hierarchical regression analysis (*N* = 653)

	Predictors	ePACI			
		1. step		2. step	
		β	*t (p)*	β	*t (p)*
*1. step*	Type of settlement (1-village, 2-city)	0.018	(0.806)	0.022	0.300 (0.764)
	Size of the settlement (1–4)	0.101	(0.173)	0.098	1.315 (0.189)
	Sex (1-male, 2-female)	0.021	(0.590)	0.022	0.567(0.571)
	Age	0.179**	(0.000)	0.131*	2.755 (0.006)
*2. step*					
	Level of education (1–6)			0.040	0.869(0.385)
	Income (1–14)			-0.012	-0.271 (0.786)
	Frequency of using the internet (0–7)			-0.089	-1.774 (0.077)
*R* ^2^		0.213		0.225	
Δ*R*^2^		0.213		0.012	
*F*		7.735**	(0.000)	4.922**	(0.000)

*p < 0.05

**p < 0.01

In the first step of the hierarchical regression, four variables enter the regression analysis: type of settlement, size of settlement, sex, and age. The first step predictors explained 21.3% of the criterion variable (ePACI), and only the variable age was a statistically significant predictor (*p* < 0.001) with positive β, meaning that older age was associated with a more conservative ePACI score.

In the second step of the hierarchical regression, three variables were added as predictors (with all the variables from the first step remaining in the analysis): level of education, income, and frequency of internet usage. This model explained 22.5% of the criterion variable (ePACI), representing only 1.2% more variance than the model with only four variables. Neither of the three new variables was statistically significant, and variable age remained the only statistically significant predictor of ePACI.

## Discussion

E-professionalism is an ever-widening phenomenon that entered the public arena almost 15 years ago [4]. However, the results of this research on the perception of professional behaviour on SM of the Croatian general population differ significantly from the perception of professionalism by Croatian students and practising HCPs [13, 18, 21].

The results show that our national representative sample had more than 75% of the respondents who use the internet every day. This finding confirms the quality of the sampling method and verifies that it’s reasonable to inquire about the behaviours of MDs and DMDs on the internet (therefore SM). Even though it was not possible to investigate the SM usage of the respondents in this research, we have relevant and contemporary data about some elements of internet use available from the Croatian Bureau of Statistics [35]. In Croatia in 2022, 86% of people had access to the internet in their household, all almost every person in age groups from 16 to 44 years old use the internet (over 98%), majority of those between 55- and 64-years old use internet (75%) and even those between 65- and 74-years old use the internet to some degree (42%). Of those who use the internet, 72% use it to access SM sites [35].

Compared to previous studies that measured the perception of e-professionalism using either the original instrument developed by White et al. [11] or its subsequent adaptations under the title “instrument to measure the perception of e-professionalism content [13, 18, 21], results from a national representative sample of the general population in Croatia show an overall more “strict” or “conservative” perception of e-professionalism of MDs and DMDs on SM. Each measured type of online behaviour was considered unprofessional by at least 60% of respondents in this study, even though previous research had shown that not all of those behaviours are considered unprofessional by the HCPs themselves [8, 11, 13, 21].

The posts that describe current relationship status are considered unprofessional by 62.6% of respondents in this study, compared to only 5% of students in White et al. research in 2013 [11], 9.6% or 9.4% of students of medicine and dental medicine in Zagreb, Croatia in 2019 and 2024 [13, 18], and MDs and DMDs in Croatia in 2021 where 24.6% of respondents perceived this as unprofessional [21]. Similar results are found in making opinionated comments about controversial issues and posts describing an interaction with a patient, while not revealing any identifying information, where a more significant proportion of the general population considered them unprofessional compared to other studies using this instrument. A picture of an individual having one alcoholic beverage should not be considered unprofessional behaviour [8], and respondents in the first creation of this measuring instrument did not consider it unprofessional either (9%) [11]. However, they are considered unprofessional by 76.5% of the general population in Croatia. This perception is also consistent with the perception of students of medicine and dental medicine in Zagreb in 2019 and 2024 [13, 18].

Endorsements of a pharmaceutical or health product without a conflict-of-interest disclosure is a behaviour that should be considered unprofessional [8] and is perceived accordingly by both the general population in Croatia (70.7%) and by MDs and DMDs in Croatia (69.7%) [21].

Status updates describing substantial alcohol consumption at a party are perceived as unprofessional by 80% of the general population in Croatia, which is similar to MDs and DMDs (80.7%) [21], but more than both student populations in previous research which were both under 70% [11, 13, 18].

Two behaviours that are considered “serious” unprofessional content [21] are perceived as unprofessional by a smaller proportion of the general population in Croatia than it was perceived in other similar studies. Posts disclosing information about a patient are considered HIPAA violations. They are perceived (correctly) as unprofessional by the student sample in White et al. (99%) [11], MDs and DMDs in Croatia (99.1%) [21], and students of medicine and dental medicine in Zagreb, Croatia (92.7% in 2019, 97.4% in 2024) [13, 18]. However, the general population in Croatia perceives this behaviour as unprofessional (90.1%), slightly less than in other studies mentioned. Similar results can be seen in perception of posts depicting illicit drug consumption, where the general population perceives it as unprofessional in a smaller proportion (90.6) than students of medicine and dental medicine [13, 18] as well as MDs and DMDs (91.1%) in Croatia [21].

With a mean of 0.59 (SD = 0.594), the ePACI index of the general population in Croatia deviates towards the conservative perception of MDs’ and DMDs’ e-professionalism. Compared to the ePACI index on the sample of MDs and DMDs in Croatia (mean = 0.36, SD = 0.433), the general population deviates even more toward the “conservative” perception [21].

The PCA extracted two underlying components that can be called “Seriously unprofessional posts” and “Professional posts”. Viskić et al. conducted PCA on the same instrument, but on a sample of MDs and DMDs in Croatia, and received four components which they called “Somewhat unprofessional behaviour”, “Professional behaviour”, “Serious unprofessional behaviour” and “Illegal and condescending behaviour” [21]. It seems that MDs and DMDs, as medical practitioners, had a finer gradient of differentiating specific behaviours than the general population. This could result from a better understanding of e-professionalism in general, through specific education (guidelines) or general socialisation in the medical/dental profession [36, 37]. Guraya et al. observed a significant improvement in the medical and dental students’ professional behaviours, after conducting an interventional workshop using the newly developed Medical Education e-Professionalism (MEeP) framework [36]. Kilic et al. described discrepancies between how doctors and non-doctors perceive SM activity [37]. Their findings suggest that non-doctors were more sensitive to perceiving posts as either unprofessional or breaching confidentiality. The reasons why doctors, patients and non-doctors perceive e-professional behaviour differently is likely due to the level of understanding of what is required of them professionally on SM [37]. The study of Maben-Feaster et al. showed that patients perceived doctors as more professional when their social media profiles contain only educational content, comparing to a mix of personal and educational content or personal content alone. This suggests that patients expect a clear separation between personal and professional profiles online [38].

Hierarchical regression analysis shows that age is the only statistically significant predictor of the ePACI index. Bivariate tests of differences between the groups showed that older people had more conservative ePACI index scores. Furthermore, those who used the internet less frequently had more conservative ePACI index scores. However, since hierarchical regression analysis showed that only age was the significant predictor, it is safe to conclude that age had a moderating role in the relationship between the frequency of internet usage and the ePACI index scores. In other words, those using the internet less frequently were older people (as can be seen from the correlation between age and internet use *r*=-0.579, *p* < 0.001), and that was the reason that usage of the internet had a significant correlation with ePACI.

It is unclear why those living in cities had a more conservative ePACI index than those living in villages (confirmed by the size of the settlement). Regression models showed that this relationship is no longer significant after the introduction of the age variable, and authors think it would be dangerous to speculate about sociocultural or other reasons for explaining this finding. Nonetheless, this could be further explored in future research, mainly through investigating what other predictors could explain the perception of e-professional behaviour of MDs and DMDs on SM. One could speculate that smaller communities have a closer relationship with their HCPs and even perceive them as more “human” with flaws that, if exhibited on SM through e-professionalism transgressions, should not or do not impede their ability to perform their professional duties. The detachment from HCPs in larger urban areas perhaps places HCPs on a higher pedestal both in ethical and professional standards, which leads to these more conservative ePACI values.

Previous research rarely compares HCPs and general population (or laypeople) differences in perception regarding SM content [39]. Gasparello et al. assessed whether orthodontists can recognize scientifically based and non-science-based posts and if their perceptions are different from general opinion (laypeople), dentistry students, and dentists (non-orthodontists) [39]. Meira et al. analysed the public perception of professional credibility and willingness to become a client, based on images posted by orthodontists on Instagram [40]. Their study population was adult laypeople, dental students and dental professionals from Brazil. Some of the themes found in the orthodontists’ social media posts were found to influence the perceptions around professional credibility and willingness to become a client, although there were differences among the participants in their study [40].

Weijs et al. [30] analysed the perception of credibility based on comments by health professionals on Facebook and observed that credibility had a major influence on the participants’ desire to become a client of these health professionals. They also concluded that positive comments related to a professional’s daily work were reflected as a perception of greater credibility [30]. Fatollah et al. conducted a cross-sectional study among 491 respondents in three U.S. cities on the impact of physicians’ social media behaviour on patients’ trust. In their study, most respondents reported they would have less trust if their physician posted racist comments online, wrote a disrespectful patient narrative, appeared intoxicated in a photograph, or wrote profanity, and respondents’ age and level of education impacted the results [41].

Freire et al. investigated the influence of the use of dental practice SM on patients when they last changed their provider [29] and observed no gender-based differences. Our study also confirms there are no gender-based differences in the perception of MDs’ and DMDs’ e-professionalism among the general population in Croatia.

Age has also been previously reported as a predictor in ePACI values and the behaviour of MDs and DMDs on SM [21]. Older HCPs have also been shown to have more conservative values of the ePACI index, and this was linked to a lack of education in SM usage and potential but also to more professional life experience and professional caution. Younger HCPs are more open to SM and have more experience and knowledge in their usage, but also have potentially not been exposed to negative backlash from professionalism transgressions, which fuels such behaviour. Previous research among different types of HCPs also emphasised age as a factor affecting the perception about e-professionalism [23, 42, 43].

Age as a predictor can also be explained because most of the education efforts in SM usage and its benefits are aimed at the younger population [44–46]. Also, in HCP education, e-professionalism education is primarily aimed at the student and resident population as part of their undergraduate or postgraduate studies [14, 47, 48]. But no research so far has been done on a representative sample of the population, nor investigated age as a predictor of the general populations’ perception of the e-professionalism of the HCPs.

These data suggest potential social implications when placed in a broader context. This is similar to the recent findings of Guraya et al. [49]. Guraya et al. emphasise the need to reclaim the concept of professionalism in the digital context [49]. According to their study, we should be aware of the wealth of generational perceptions surrounding the concept of e-professionalism, including digital literacy and the competency gap [49]. Are digital natives digitally competent? Young HCPs, although called “digital natives,” are helpless when faced with permanency, mixing, interpolation, and shaping of digital content [50]. It is not clear whether we are witnessing the liberalization of the perception of new generations (based on our findings of perception differences based on age) or if this is another manifestation of the digital divide and resistance of older generations to new technologies [51].

Just as there are dangers and benefits of SM, there are also risks and benefits at both ends of the spectrum when it comes to the liberalization of perception towards e-professionalism in HCPs. On the one hand, liberal perception opens opportunities for greater self-expression for HCPs, justifying their more active engagement in using SM. On the other hand, it carries an increased risk of violating patient privacy and mishandling confidential data.

The results of this study can provide insights to creators of medical education curricula on how the general population perceives MDs’ and DMDs’ activities on SM. The findings indicate that a portion of the population, particularly the older demographic, has the least understanding of MDs’ and DMDs’ activities on SM, and they are more inclined to perceive their posts as unprofessional, even more so than their colleagues and other healthcare professionals. The need to incorporate e-professionalism education into medical education curricula and equip doctors to manage their online image actively and effectively, especially signifying the importance of tailored educational strategies to ensure HCPs uphold high standards while practicing in the digital realm has been recognized in previous research [23, 49]. But HCPs should be aware that the truth is in the eye of the beholder, hence, who the beholder is depends on how their online activity will be assessed in terms of professionalism.

### Strengths

The greatest strength of this study lies in the quality of the sample. In addition to being representative, the sample is also robust, consisting of 1,000 respondents, a size that aligns with internationally established surveys such as the *Eurobarometer Data Service* (*Standard* and *Special Eurobarometer surveys*) [52], as well as the European Quality of Life Survey (EQLS) by Eurofound [53], all of which use the same sample size for Croatia. This research thus represents a comprehensive approach that emphasizes the perception of the general population on the e-professionalism of MDs and DMDs, based on a probabilistic representative sample from one country. To the best of the authors’ knowledge, this is the first study to explore this topic using such a sample, addressing a notable gap in the field of e-professionalism research.

Another strength of this study is the use of a validated instrument for measuring the perception of unprofessional content, which enables the comparison of results across different populations where the study was conducted.

### Limitations

The first limitation of this study is the use of the ePACI instrument on a population for which the instrument was not initially designed. This instrument was created to measure the perceptions among HCPs, and there is a possibility that it may require some adaptation. This study has shown that there were no items in the instrument that the general population did not understand or could not respond to, however, in future research, it is necessary to attempt to ascertain if there is any missing content that would be necessary for the general population but was not needed for the HCP population.

The second limitation of this study is the inability to measure whether the respondents are users of SM or not. This limitation arises from the research methodology employed, specifically the “omnibus” type of research conducted in collaboration with the Ipsos Agency, which has a fixed number of sociodemographic variables. Therefore, in this study, it was not possible to capture additional variables related to internet usage habits, including specific details about SM usage. A recommendation for future research is to incorporate the measurement of SM usage, to rerun the regression model exclusively on SM users and to compare the results.

The third limitation of this research relates to missing values. Since some respondents did not answer all the questions in the perception instrument, a smaller subset of the collected data was used for analysis (788/1000). In this study, it was not possible to investigate why respondents chose not to answer specific questions. The distinction between a lack of understanding of the question and refusal to answer a question is essentially the difference between “missing at random” (MAR) and “missing not at random” (MNAR) [54]. However, considering that missing values were equally distributed among the items, and there was no item in the perception measurement instrument without at least one missing value, the authors believe that missing data in this study is MAR and, therefore, not problematic.

## Conclusion

This research presents the results of the perception of e-professionalism among the general population in Croatia regarding the posts of MDs and DMDs on SM. The results, both at the individual item level and on the ePACI index, indicate that the general population holds a strict or “conservative” perception of e-professionalism. This suggests that a significant number of posts on the MDs’ and DMDs’ profiles might be perceived as unprofessional.

Hierarchical regression analysis revealed that age is the only significant predictor of the ePACI index for the general population, with older individuals demonstrating a more “conservative” perception of the professionalism of doctors’ posts on SM. These results provide a novel perspective on the perception of e-professionalism among MDs and DMDs, as previous findings have primarily focused on how HCPs perceive e-professionalism. The results of this research on the sample of the general population fill in the previous knowledge gaps on e-professionalism perception.

Given that the general population is more inclined to perceive posts as unprofessional compared to MDs and DMDs themselves, there is a need for continuous and focused education for doctors to maintain their e-professionalism that also includes the perception of the general population.

## Supplementary Information

Supplementary Material 1.

## Data Availability

The datasets used and/or analysed during the current study are available from the corresponding author on reasonable request.

## Abbreviations

DMD: Doctor of Dental Medicine
ePACI: e–Professionalism Assessment Compatibility Index
HCP: Health Care Professional
HIPAA: The Health Insurance Portability and Accountability Act
MAR: Missing at random
MEeP: Medical Education e–professionalism
MD: Medical Doctor
MNAR: Missing not at random
PCA: Principal Component Analysis
SM: Social Media
SMePROF: Social Media e–Professionalism

## References

[1] Masson F, Ross E. Professionalism and social media in the 21st century. In: Geraint T, editor. Professionalism: Perspectives and Practices of the 21st Century. New York: Nova Science; 2017.

[2] Greysen SR, Johnson D, Kind T, Chretien KC, Gross CP, Young A, et al. Online Professionalism Investigations by State Medical Boards: First, Do No Harm. Ann Intern Med. 2013;158:124. 23318312 10.7326/0003-4819-158-2-201301150-00008

[3] Staud SN, Kearney RC. Social Media Use Behaviors and State Dental Licensing Boards. J Dent Hyg. 2019;93:37–43. 31182567

[4] Cain J, Romanelli F. E-professionalism: a new paradigm for a digital age. Currents Pharm Teach Learn. 2009;1:66–70.

[5] Londyn Robinson article says photos of vasc surgeons in a. provocative pose wearing bikini is unprofessional. Twitter. 2020. Jul 3. Available from: https://twitter.com/londyloo/status/1286361257872896000. Accessed 12 Oct 2023.

[6] Annear S, #MedBikini. Here’s why health care professionals are posting photos of themselves in bathing suits.- The Boston Globe. 2020. Jun 24. Available from: https://www.bostonglobe.com/2020/07/24/metro/medbikini-heres-why-healthcare-professionals-are-posting-photos-themselves-bathing-suits-social-media/. Accessed 12 Oct 2023.

[7] Laughney C. What you need to know about the infamous #MedBikini Study. Medium. 2020. Apr 10, [2023-10-12]. Available from: https://medium.com/beingwell/what-you-need-to-know-about-the-infamous-medbikini-study-1d814e1ebe44. Accessed 12 Oct 2023.

[8] Vukušić Rukavina T, Machala Poplašen L, Majer M, Relić D, Viskić J, Marelić M. Defining Potentially Unprofessional Behavior on Social Media for Health Care Professionals: Mixed Methods Study. JMIR Med Educ. 2022;8:e35585. 35758605 10.2196/35585 PMC9399843

[9] Aboalshamat K, Alkiyadi S, Alsaleh S, Reda R, Alkhaldi S, Badeeb A, et al. Attitudes toward Social Media among Practicing Dentists and Dental Students in Clinical Years in Saudi Arabia. Open Dentistry J. 2019;13:143–9.

[10] Mani SA, Uma E, John J, Nieminen P. Perceptions of professional social media interaction with patients and faculty members – a comparative survey among dental students from Malaysia and Finland. BMC Med Educ. 2023;23:384. 37231460 10.1186/s12909-023-04359-1 PMC10214545

[11] White J, Kirwan P, Lai K, Walton J, Ross S. Have you seen what is on Facebook?’ The use of social networking software by healthcare professions students. BMJ Open. 2013;3:e003013. 10.1136/bmjopen-2013-003013 PMC3731743 23883886

[12] Dobson E, Patel P, Neville P. Perceptions of e-professionalism among dental students: a UK dental school study. Br Dent J. 2019;226:73–8. 30631197 10.1038/sj.bdj.2019.11

[13] Viskić J, Jokić D, Marelić M, Machala Poplašen L, Relić D, Sedak K, et al. Social media use habits, and attitudes toward e-professionalism among medicine and dental medicine students: a quantitative crosssectional study. Croat Medl J. 2021;62:569–79. 10.3325/cmj.2021.62.569 PMC8771237 34981689

[14] Soubra R, Hasan I, Ftouni L, Saab A, Shaarani I. Future healthcare providers and professionalism on social media: a cross-sectional study. BMC Med Ethics. 2022;23:4. 35057787 10.1186/s12910-022-00742-7 PMC8781471

[15] Alzahrani AK, Banaser AH, Alsulami RR, Alluqmani YA, Althubyani GS, Al Luhaybi FH, et al. Comparative assessment of attitudes among medical and dental professionals in Saudi Arabia toward e-professionalism using the SMEPROF-S scale. J Family Med Prim Care. 2023;12:1137–44. 37636193 10.4103/jfmpc.jfmpc_2192_22 PMC10451607

[16] Guraya SS, Guraya SY, Yusoff MSB. Preserving professional identities, behaviors, and values in digital professionalism using social networking sites; a systematic review. BMC Med Educ. 2021;21:381. 34247617 10.1186/s12909-021-02802-9 PMC8273947

[17] Kitsis EA, Milan FB, Cohen HW, Myers D, Herron P, McEvoy M, et al. Who’s misbehaving? Perceptions of unprofessional social media use by medical students and faculty. BMC Med Educ. 2016;16:67. 26887561 10.1186/s12909-016-0572-x PMC4757980

[18] Relić D, Marelić M, Viskić J, Machala Poplašen L, Majer M, Sedak K, Vukušić Rukavina T. Exploring changes in the perception of e-professionalism among medical and dental students: a quantitative cross-sectional study. Croat Med J. 2024;65(1):43–50. 38433511 10.3325/cmj.2024.65.43 PMC10915766

[19] Marelić M, Viskić J, Poplašen LM, Relić D, Jokić D, Rukavina TV. Development and validation of scale for measuring attitudes towards e-professionalism among medical and dental students: SMePROF-S scale. BMC Med Educ. 2021;21:445. 34425792 10.1186/s12909-021-02879-2 PMC8381479

[20] Smailhodzic E, Hooijsma W, Boonstra A, Langley DJ. Social media use in healthcare: A systematic review of effects on patients and on their relationship with healthcare professionals. BMC Health Serv Res. 2016;16:442. 27562728 10.1186/s12913-016-1691-0 PMC5000484

[21] Viskić J, Marelić M, Machala Poplašen L, Vukušić Rukavina T. Differences between doctors of medicine and dental medicine in the perception of professionalism on social networking sites: the development of the e-professionalism assessment compatibility index (ePACI). BMC Med Ethics. 2022;23:129. 36474221 10.1186/s12910-022-00870-0 PMC9727956

[22] Surani Z, Hirani R, Elias A, Quisenberry L, Varon J, Surani S, et al. Social media usage among health care providers. BMC Res Notes. 2017;10:654. 29187244 10.1186/s13104-017-2993-y PMC5708107

[23] Vukušić Rukavina T, Viskić J, Machala Poplašen L, Relić D, Marelić M, Jokic D, et al. Dangers and Benefits of Social Media on E-Professionalism of Health Care Professionals: Scoping Review. J Med Internet Res. 2021;23:e25770. 34662284 10.2196/25770 PMC8663533

[24] Pagoto S, Schneider KL, Evans M, Waring ME, Appelhans B, Busch AM, et al. Tweeting it off: characteristics of adults who tweet about a weight loss attempt. J Am Med Inf Assoc. 2014;21:1032–7. 10.1136/amiajnl-2014-002652 PMC4215051 24928175

[25] Chirumamilla S, Gulati M. Patient Education and Engagement through Social Media. Curr Cardiol Rev. 2021;17:137–43. 31752656 10.2174/1573403X15666191120115107 PMC8226210

[26] Benetoli A, Chen TF, Aslani P. How patients’ use of social media impacts their interactions with healthcare professionals. Patient Educ Couns. 2018;101:439–44. 28882545 10.1016/j.pec.2017.08.015

[27] Farsi S, Sabbahi A, Sait D, Kabli R, Abduljabar G. Ethical Use of Social Media and Sharing of Patient Information by Medical Students at a University Hospital in Saudi Arabia: Cross-Sectional Survey. JMIR Med Educ. 2025;11:e57812. 40127453 10.2196/57812 PMC11957465

[28] Poplašen LM, Marelić M, Vukušić Rukavina T. Differences between Doctors of Dental Medicine and Doctors of Medicine Awareness of Their Online Image and Perception Concerns: a Quantitative Cross-Sectional Study. Acta Stomatol Croat. 2024;58(3):291–304. 39492866 10.15644/asc58/3/8 PMC11526828

[29] Freire Y, Sánchez MG, Suárez A, Joves G, Nowak M, Díaz-Flores García V. Influence of the use of social media on patients changing dental practice: a web-based questionnaire study. BMC Oral Health. 2023;23:365. 37277765 10.1186/s12903-023-03078-9 PMC10243041

[30] Weijs C, Coe J, Desmarais S, Majowicz S, Jones-Bitton A. Effects of Mock Facebook Workday Comments on Public Perception of Professional Credibility: A Field Study in Canada. J Med Internet Res. 2019;21:e12024. 30998223 10.2196/12024 PMC6495291

[31] Ipsos. Omnibus. Zagreb. Ipsos; c2023. Available from: https://www.ipsos.com/hr-hr/omnibus-0. Accessed 24 Oct 2023.

[32] QuestionPro Survey Software. Omnibus survey definition:surveyanalytics - online survey software. San Francisco: Survey Analytics LLC; c2023. Available from: https://www.surveyanalytics.com/omnibus-survey-definition.html. Accessed 25 Oct 2023.

[33] Croatian Bureau of Statistics. The census of population, households and dwellings in 2011. [Croatian] Available from: https://web.dzs.hr/Hrv/censuses/census2011/censuslogo.htm. Accessed 24 Oct 2023.

[34] Croatian Bureau of Statistics. The census of population, household and dwellings in 2021. [Croatian] Available from: https://popis2021.hr/. Accessed 24 Oct 2023.

[35] Croatian Bureau of Statistics. ZTI-2022-1-2 usage of Information and Communication Technologies (ICT) in Households and by Individuals. 2022. Available from: https://podaci.dzs.hr/2022/en/29623. Accessed 30 Aug 2023.

[36] Guraya SS, Yusoff MSB, Rashid-Doubell F, Harkin DW, Al-Amad SH, Fredericks S, et al. Changing Professional Behaviors in the Digital World Using the Medical Education e-Professionalism (MEeP) Framework-A Mixed Methods Multicentre Study. Front Med (Lausanne). 2022;9:846971. 35425778 10.3389/fmed.2022.846971 PMC9004460

[37] Kilic Y, Chauhan D, Avery P, Horwood N, Nakov R, Disney B, et al. The public’s attitude towards doctors’ use of Twitter and perceived professionalism: an exploratory study. Clin Med (Lond). 2021;21:e475–9. 34507932 10.7861/clinmed.2021-0357 PMC8439510

[38] Maben-Feaster RE, Stansfield RB, Opipari A, Hammoud MM. Evaluating Patient Perspectives of Provider Professionalism on Twitter in an Academic Obstetrics and Gynecology Clinic: Patient Survey. J Med Internet Res. 2018;20(3):e78. 29530838 10.2196/jmir.8056 PMC5869178

[39] Gasparello GG, Mota-Júnior SL, Hartmann GC, Berlesi AH, Acciaris F, Berretta LM, Pithon MM, Tanaka O. Orthodontics social media, perceptions of science- and non-science-based posts among orthodontists, dentists, students and laypeople. PLoS ONE. 2023;18(9):e0286927. 37773974 10.1371/journal.pone.0286927 PMC10540967

[40] Meira TM, Prestes J, Gasparello GG, Antelo OM, Pithon MM, Tanaka OM. The effects of images posted to social media by orthodontists on public perception of professional credibility and willingness to become a client. Prog Orthod. 2021;22(1):7. 33682012 10.1186/s40510-021-00353-9 PMC7937582

[41] Fatollahi JJ, Colbert JA, Agarwal P, Lee JL, Lehmann EY, Yuan N, Lehmann LS, Chretien KC. The impact of physician social media behavior on patient trust. AJOB Empir Bioeth. 2020;11(2):77–82. 10.1080/23294515.2019.1678533 31663810

[42] Koo K, Bowman MS, Ficko Z, Gormley EA. Older and wiser? Changes in unprofessional content on urologists’ social media after transition from residency to practice. BJU Int. 2018;122:337–43. 29694713 10.1111/bju.14363

[43] Ryan-Blackwell G. Achieving shared values: A mixed methods study and multi-method model of how to effectively educate nurses about e-professionalism. J Nurs Educ Pract. 2023;13:47–58.

[44] Xie B, Watkins I, Golbeck J, Huang M. Understanding and Changing Older Adults’ Perceptions and Learning of Social Media. Educ Gerontol. 2012;38:282–96. 22639483 10.1080/03601277.2010.544580 PMC3358790

[45] Hruska J, Maresova P. Use of Social Media Platforms among Adults in the United States—Behavior on Social Media. Societies. 2020;10:27.

[46] Chan-Olmsted SM, Cho M, Lee S. User Perceptions of Social Media: A Comparative Study of Perceived Characteristics and User Profiles by Social Media. Online J Communication Media Technol. 2013;3:149–78.

[47] Kaczmarczyk JM, Chuang A, Dugoff L, Abbott JF, Cullimore AJ, Dalrymple J, et al. e-Professionalism: a new frontier in medical education. Teach Learn Med. 2013;25:165–70. 23530680 10.1080/10401334.2013.770741

[48] Guckian J, Utukuri M, Asif A, Burton O, Adeyoju J, Oumeziane A, Chu T, Rees EL. Social media in undergraduate medical education: A systematic review. Med Educ. 2021;55(11):1227–41. 33988867 10.1111/medu.14567

[49] Guraya SS, Guraya SY, Rashid-Doubell F, Fredericks S, Harkin DW, Bin Mat Nor MZ, Bahri Yusoff MS. Reclaiming the concept of professionalism in the digital context: a principle-based concept analysis. Ann Med. 2024;56(1):2398202. 39263743 10.1080/07853890.2024.2398202 PMC11395874

[50] Guraya SS, Rashid-Doubell F, Harkin DW, Guraya SY. Mission-driven e-professionalism in the medical field: shaping digital identity and virtual engagement. Front Med (Lausanne). 2024;11:1276839. 38585143 10.3389/fmed.2024.1276839 PMC10996440

[51] Ahn J. Digital Divides and Social Network Sites: Which Students Participate in Social Media? J Educational Comput Res. 2011;45:147–63.

[52] GESIS, Leibniz Institute for the Social Science. Eurobarometer Dana Service: Dana and Documentation: Standard & Special EB: Population, countries & regions. Mannheim: GESIS. Available from: https://www.gesis.org/en/eurobarometer-data-service/data-and-documentation/standard-special-eb/population-countries-regions. Cited 24 Apr 2025.

[53] Eurofound. Methodology: sample size. Dublin: European Foundation for the improvement of living and working conditions. Available from: https://www.eurofound.europa.eu/sl/methodology-sample-size. Cited 24 Apr 2025.

[54] Tabachnick BG, Fidell LS. Using Multivariate Statistics. 6th ed. London: Pearson Education; 2013.

