# Supplementary material for: Perception of e-professionalism of doctors of medicine and doctors of dental medicine among the general population in Croatia
Source: BMC Med Ethics. 2026 Feb 28;27:68. doi: 10.1186/s12910-026-01427-1 (PMC13059232; doi:10.1186/s12910-026-01427-1)
Supplement: Supplementary file 1 — Supplementary Material 1. [file 12910_2026_1427_MOESM1_ESM.docx]

The questionnaire used in this study has two parts: (1) the instrument: “Perception of e-professionalism of doctors of medicine and doctors of dental medicine” and (2) the sociodemographic characteristics of the respondents.

***I. The instrument: “Perception of e-professionalism of doctors of medicine and doctors of dental medicine”***

**1. Below are some of the possible types of posts that a medical doctor/doctor of dental medicine could make on their PRIVATE social media accounts. Please respond which of the following types of posts you consider unprofessional?**

|  | **Yes, I consider it unprofessional** | **No, I do not consider it unprofessional** | **I don't know/No answer** |
| --- | --- | --- | --- |
| 1. A picture of an individual having one alcoholic beverage. |  |  |  |
| 1. Pictures of an individual clearly behaving drunkenly. |  |  |  |
| 1. Status updates describing substantial alcohol consumption at a party. |  |  |  |
| 1. Posts depicting illicit drug consumption. |  |  |  |
| 1. Posts disclosing information about a patient/client. |  |  |  |
| 1. Photos of a patient/client. |  |  |  |
| 1. Posts describing an interaction with a patient/client, while not revealing any identifying information. |  |  |  |
| 1. Swearing or foul language. |  |  |  |
| 1. Obscene gestures in photos (the middle finger, etc.). |  |  |  |
| 1. Petty criminal activity. |  |  |  |
| 1. Endorsements of a pharmaceutical or health product without a conflict-of-interest disclosure. |  |  |  |
| 1. Posts involving overt sexual content. |  |  |  |
| 1. Posts containing partial nudity. |  |  |  |
| 1. Displaying your current relationship status. |  |  |  |
| 1. Displaying membership in online groups dealing with controversial issues. |  |  |  |
| 1. Making opinionated comments about controversial issues. |  |  |  |
| 1. Attitudes of superiority or condescending behaviour (assumed because of professional status). |  |  |  |

***II. Sociodemographic characteristics of the respondents***

**2. Type of settlement**

1. Village
2. City

**3. Sex**

1. Male
2. Female

**4. Size of the settlement**

1. Up to 2000 inhabitants
2. 2,001 – 10,000 inhabitants
3. 10, 001 – 75,000 inhabitants
4. 75,001 and more inhabitants

**5. Age** ___________

**6. Select from this card the last school you graduated from.**

1. Elementary school or lower
2. Vocational school
3. Gymnasium
4. Undergraduate study / First degree of the faculty (Bachelor)
5. Graduate study / Faculty / Academy / College
6. Postgraduate studies (specialist, master’s, PhD)
7. Don’t know/Without an answer

**7. How often do you personally use the internet?**

1. Every day
2. Several times a week
3. Once a week
4. Several times a month
5. Once a month
6. Rarely
7. I do not use the internet

**8. Choose the letter next to the amount that corresponds to your total personal income for the past month.**

1. No personal income last month
2. Up to HRK 1,000 (132.72€)
3. From HRK 1,001 (132.86€) to HRK 2,000 (265.45€)
4. From HRK 2001 (265.58€) to HRK 3000 (398.17€)
5. From HRK 3,001 (398.30€) to HRK 4,000 (530.89€)
6. From HRK 4,001 (531.02€) to HRK 5,000 (663.61€)
7. From HRK 5,001 (663.75€) to HRK 6,000 (796.34€)
8. From HRK 6,001 (796.47€) to HRK 7,000 (929.06€)
9. From HRK 7,001 (929.19€) to HRK 8,000 (1061.78€)
10. From HRK 8,001 (1061.92€) to HRK 9,000 (1194.51€)
11. From HRK 9,001 (1194.64€) to HRK 10,000 (1327.23€)
12. From HRK 10,001 (1327.36€) to HRK 11,000 (1459.95€)
13. From HRK 11,001 (1460.08€) to HRK 12,000 (1592.67€)
14. From HRK 12,001 (1592.81€) and more
15. Don’t know/Refuse to answer
